# The Role of Beclin 1-Dependent Autophagy in Cancer

**DOI:** 10.3390/biology9010004

**Published:** 2019-12-22

**Authors:** Silvia Vega-Rubín-de-Celis

**Affiliations:** 1 Institute for Cell Biology (Tumorforschung), University Hospital Essen, 45122 Essen, Germany; Silvia.Vega.Rubindecelis@gmail.com; Tel.: +49-0201-723-3941; 2 German Cancer Consortium (DKTK) at Essen-Düsseldorf, 445122 Essen, Germany

**Keywords:** Beclin 1, autophagy, cancer

## Abstract

Autophagy (*self-eating*) is an intracellular degradation process used by cells to keep a “clean house”; as it degrades abnormal or damaged proteins and organelles, it helps to fight infections and also provides energy in times of fasting or exercising. Autophagy also plays a role in cancer, although its precise function in each cancer type is still obscure, and whether autophagy plays a protecting (through the clearing of damaged organelles and protein aggregates and preventing DNA damage) or a promoting (by fueling the already stablished tumor) role in cancer remains to be fully characterized. Beclin 1, the mammalian ortholog of yeast Atg6/Vps30, is an essential autophagy protein and has been shown to play a role in tumor suppression. Here, an update of the tumorigenesis regulation by Beclin 1-dependent autophagy is provided.

## 1. Introduction

**Figure 1:**
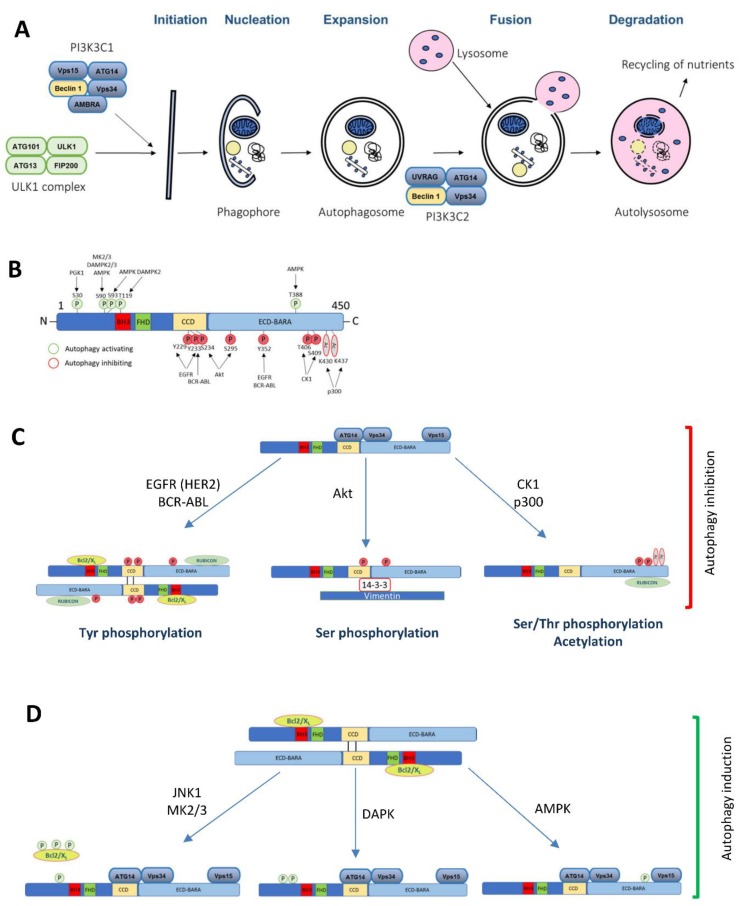
The Beclin 1 function in autophagy and the role of its post-translational modifications implicated in cancer. (**A**) A diagram of the macroautophagy pathway highlighting the complexes where Beclin 1 is involved in. (**B**) A schematic illustration of the Beclin 1 post-translational modifications implicated in cancer and its consequences in autophagy (**C**) inhibition or (**D**) induction.

Human *BECN1* (*Beclin 1* autophagy related gene) is located on chromosome 17q21, and monoallelic deletions of that region are found in up to 50% of breast cancers, 75% of ovarian cancers and 40% of prostate cancers [1]. *BECN1* is a haploinsufficient tumor suppressor [2,3,4] and although it is not frequently mutated in cancers, *Becn1*^+/−^ mice show an increased incidence of spontaneous tumors, including mammary carcinoma [3,4,5]. Beclin 1, the protein encoded by the *BECN1* gene, is essential for autophagy [2]. Autophagy is an intracellular degradation process in which the engulfment of cytoplasmic and organelle components by double-membraned vesicles, autophagosomes, leads to their degradation into the lysosome. Autophagy is an evolutionary conserved pathway that is implicated in many physiological (starvation, low oxygen, removal of damaged organelles, exercise, removal of intracellular pathogens, etc.) and pathological conditions, including protection against diseases, such as cancer [6] (Figure 1A). This review will focus on the role of Beclin 1-dependent autophagy in cancer.

## 2. Beclin 1 Structure and Its Function in Autophagy

Mammalian Beclin 1 was first discovered in a yeast-two hybrid screen of Bcl-2 (B-cell lymphoma 2) interactors [7], and further studies described Beclin 1 as the first mammalian homolog of the yeast autophagy gene *ATG6/VPS30* [2]. Beclin 1 is a 60 kDa protein that contains an intrinsically disordered N-terminal region (amino acids 1–130) [8,9] and four different domains: Bcl-2-homology 3 (BH3; encompassing amino acids 105–130) [10], a flexible helix domain (amino acids 141–171) [11], a coiled coil domain (CCD; amino acids 175–264) [12], and an evolutionary conserved domain (ECD; amino acids 248–337) [13], also termed as a β-α repeated autophagic-specific domain (BARA; amino acids 265–450) [13,14] (Figure 1B).

Mammalian Beclin 1 functions as an allosteric modulator of the class III phosphatidylinositol 3-kinase (PI3KC3) complexes (PI3KC3-C1 and PI3KC3-C2) that generate phosphatidylinositol 3-phosphate (PI3P) (Figure 1A). PI3KC3-C1 functions in the autophagic vesicle enucleation, at the initial stages of the autophagosome formation and contains Beclin 1, Vps34, Vps15, Atg14, and Ambra [15,16,17,18,19,20]. PI3KC3-C2 is involved in the autophagolysosomal maturation and contains Beclin 1, Vps34, Vps15, and UVRAG (UV Radiation Resistance Associated Gene Protein). Rubicon (RUN domain and cysteine-rich containing, Beclin 1-interacting protein) interacts with the PI3KC3-C2 to inhibit its lipid kinase activity [15,16,21]. Besides autophagy, Beclin 1 is also involved in other pathways, including endocytic trafficking [15,16,17,18,21] or LC3 (microtubule-associated protein 1 light chain 3)-associated phagocytosis [19].

Bcl-2 and Bcl-xL are major regulators of the autophagy activity through Beclin 1 modulation [20]. It was shown that they inhibit autophagy through its binding to the BH3 domain of Beclin 1, stabilizing Beclin 1 homodimerization and further inactivation due to its inability to bind other binding partners within the PI3KC3 complex [7]. Numerous stimuli have been shown to regulate the Bcl-2/Bcl-XL interaction with Beclin 1 to regulate autophagy induction in stress conditions, such as starvation or hypoxia. The phosphorylation of both Bcl-2/Bcl-xL and Beclin 1 is a major regulator of this interaction. Under nutrient starvation, JNK1 (c-Jun amino terminal kinase 1) phosphorylates Bcl-2 at T69, S70, and S97, promoting the dissociation of Bcl-2-Beclin 1 and the subsequent autophagy activation [22]. Beclin 1 can also be phosphorylated at T119 in its BH3 domain by DAPK (Death associated protein kinase) or ROCK1 (Rho kinase 1) [23,24], promoting its dissociation from Bcl-2 and inducing autophagy through Beclin 1 binding to the Vps34 complex (Figure 1B,D). Other regulators of the Bcl-2-Beclin 1 interaction are the stress responsive kinases MK2 and MK3. Upon starvation, MK2/3 phosphorylate Beclin 1 at S90, disrupting its binding with Bcl-2 and inducing autophagy [25] (Figure 1B,D). Additionally, it was shown that Mst1 (mammalian Ste20-like kinase 1) is also able to phosphorylate Beclin 1 at T108 (within its BH3 domain), promoting the interaction of Bcl-2 and Beclin 1 and therefore inhibiting autophagy [26]. Some of these modifications will be discussed in more detail in the following sections.

## 3. Mutation Status of Beclin 1 in Cancer

Several studies suggest that the *BECN1* gene is not heavily mutated in cancer. Mutations in gastric (p.N8K, p.R389C) and colorectal (p.P350R) cancers have been reported [27], and around 0.5% of patients were found to have *BECN1* mutations across different cancer types [28]. Data from the COSMIC (Catalog of Somatic Mutations) database revealed a similar percentage, with small intestine (1.82%), ovary (1.03%), and skin (0.86%) cancers being among the most highly mutated tumor entities, and 50% of the mutations are missense or synonymous substitutions.

Interestingly, the *BECN1* gene is monoallellicaly deleted in 40%–75% of breast, ovarian, and prostate cancers [1,2,29]. Due to the close proximity of the *BRCA1* (breast cancer 1, early onset) and the *BECN1* gene at the 17q21 chromosome, it was postulated that *BECN1* deletions are rather a passenger event [28]. *BRCA1* is frequently mutated in familial cases of breast and ovarian cancer, being relatively rare in sporadic cancers, and it is a classical tumor suppressor, as only one copy is sufficient to maintain its function. By contrast, the loss of just one allele of *BECN1* is sufficient to induce tumorigenesis [3,4], and therefore it is classified as a haploinsufficient tumor suppressor. Furthermore, a survival analysis on the METABRIC (Molecular Taxonomy of Breast Cancer International Consortium) dataset showed that a worse survival probability was associated with the lower *BECN1* but not with the *BRCA1* mRNA expression in all breast cancer types [30], indicating that in sporadic breast cancers, *BECN1* is a driver rather than a passenger event.

Decreased Beclin 1 proteins have been reported in breast cancers compared to normal tissue [2,31], as well as in other cancer entities, such as ovarian carcinomas [32,33], colon cancer [34,35], non-small cell lung cancer [36,37], cholangiocarcinoma [38], gastric cancer [39,40], and renal cell carcinoma [41]. A poorer prognosis of patients expressing low Beclin 1 levels has also been reported in multiple tumor entities, such as breast [30,42], ovarian [32,33], oral [43], gastric [39,40], and renal cell carcinoma [41].

Taken together, these data suggest that a decreased Beclin 1 expression occurs frequently in tumors when compared to normal samples, and it also suggest that lower Beclin 1 levels usually correlate with a worse prognosis in multiple cancer types.

## 4. Beclin 1 Modifications Involved in Cancer

The Beclin 1 function is regulated at different levels, including post-translational modifications, such as phosphorylation, ubiquitination or acetylation, dynamic interactome changes, modifications driving a different subcellular localization, or at the transcriptional level [44,45,46,47,48]. Although multiple post-translational modifications have been shown to modify Beclin 1 and its function in autophagy [49,50], the main focus of this section is on the cancer-related regulatory pathways that modulate autophagy and tumorigenesis through Beclin 1 (Figure 1B–D; Table 1).

### 4.1. Autophagy-Activating Modifications on Beclin 1

#### 4.1.1. MK2/3

Mitogen-activated protein kinases (MAPKs) are serine/threonine kinases involved in multiple cellular pathways to regulate a variety of processes, such as gene expression, proliferation, embryogenesis, mitosis, and apoptosis [51]. Beclin 1 is phosphorylated at S90 by members of the p38α MAPK family MK2 and MK3 under starvation conditions. Bcl-2 binding to Beclin 1 prevents the MK2/3 access to Beclin 1, and such binding is released in starvation conditions, when JNK1 phosphorylates Bcl-2, releases it from Beclin 1 [22], and keeps Beclin 1 S90 accessible to phosphorylation. Interestingly, S90 phosphorylation is essential for the Beclin 1 tumor-suppressing function, as shown in breast cancer MCF-7 xenografts [25], since a non-phosphorylatable mutant of Beclin 1 (Beclin 1 S90A) did not recapitulate the tumor suppression function of the wild-type Beclin 1.

#### 4.1.2. DAPK2/3

The death-associated protein kinase (DAPK) family contains Serine/Threonine Ca^+2^/calmodulin-regulated kinases involved in regulating apoptotic cell death, the immune system, cytoskeletal dynamics, and autophagy-mediated cell death. It contains three highly related family members (DAPK1, 2, and 3) that share around 80% homology at their kinase domains [52]. DAPK family members were postulated to act as tumor suppressors, as a decreased expression was found in several cancer entities (including bladder, breast, renal cell carcinoma, lymphoma, prostate, and brain tumors) due to epigenetic silencing through promoter methylation and also to loss of heterozygosity [53].

On an early report, Zalckvar and colleagues showed that DAPK binds to Beclin 1 and phosphorylates it at T119, disrupting the Bcl-2 and Bcl-XL binding and activating autophagy [24], although it was unclear what family member was responsible for this modification. DAPK2 is regulated by AMPK (AMP activating protein kinase), and AMPK-induced phosphorylation of DAPK2 at S289 leads to DAPK2 activation, the phosphorylation of Beclin 1 at T119, the disruption of the Beclin 1-Bcl-2/XL binding, and autophagy activation. However, the significance of these modifications by AMPK/DAPK2 *in vivo* or in cancer needs further investigation [54]. Another member of the family, DAPK3, was shown to phosphorylate Beclin 1 at S90, and this phosphorylation event is counteracted by PP2A (Protein Phosphatase 2A) [55]. Although the relevance in cancer of the Beclin 1 phosphorylation/dephosphorylation by the DAPK-family members and PP2A needs to be explored, these data indicate a potential coordinated mechanism of S90 phosphorylation by multiple kinases including AMPK, MK2/3, and DAPK family members. Due to the importance of this residue in autophagy modulation and tumorigenesis [25], and to its complex control, it would be interesting to further investigate the fine-tuning regulation of this site and other potential players implicated on it, including phosphatases.

#### 4.1.3. AMPK

AMPK is a kinase of the Liver kinase B1 (LKB1) signaling pathway that is activated upon ATP depletion in the cell. It plays important roles in regulating cell growth, glucose and lipid metabolism, transcription, and cell polarity [56]. AMPK was shown to regulate autophagy by phosphorylating ULK1 (unc-51 like the autophagy activating kinase 1) at S467, S555, T574, and S637 [57]. In starvation conditions, AMPK also phosphorylates Beclin 1 at S93, S96 [58], and T388 [59], but the role of these phosphorylation events in tumorigenesis needs to be explored. Beclin 1 phosphorylation by AMPK seem to have other effects not related with autophagy induction. Thus, Beclin 1 phosphorylation by AMPK at S90, S93, S96 regulates ferroptosis, an iron-dependent cell death, upon binding to SLC7A11 (solute carrier family 7 member 11). Indeed, Beclin 1 overexpression or Tat-Beclin 1 treatment increases ferroptosis cell death and decreases tumor growth *in vivo* [60]. However, the direct effects of these serine phosphorylation events by AMPK require further investigation.

#### 4.1.4. PGK1

Phosphoglycerate kinase 1 (PGK1) is a glycolytic enzyme that catalyzes the transfer of the phosphate from the 1 position of 1,3-diphosphoglycerate to ADP, generating 3-phosphoglycerate and ATP, and has been shown to be overexpressed in multiple cancer types [61]. Upon ARD1-dependent acetylation at K388, PGK1 binds to Beclin 1 and phosphorylates it at S30, leading to the activation of the PI3KC3 complex and the induction of autophagy [62]. Xenograft tumors generated from U87 glioblastoma cells showed that both Beclin 1 depletion, as well as the expression of a Beclin 1 S30A mutant, decreased tumorigenesis, and the reconstitution with wild-type Beclin 1 increased it. Furthermore, the reconstitution of the wild-type form of Beclin 1 induced autophagy, as determined by decreased p62 and increased Ki67 levels by immunohistochemistry (IHC). An analysis of patient samples with elevated levels of phosphorylated Beclin 1 S30 showed a significantly lower percent survival than for those patients with a low Beclin 1 S30 phosphorylation. Taken together, these data suggest that, in this context, autophagy activation acts as a pro-survival mechanism and favors tumor development and a poorer prognosis in patients.

### 4.2. Autophagy-Inhibiting Modifications on Beclin 1

#### 4.2.1. EGFR Family

The epidermal growth factor receptor (EGFR) is an oncogenic transmembrane receptor tyrosine kinase family of four receptors (EGFR/ERBB1/HER1, ERBB2/HER2, ERBB3/HER3, and ERBB4/HER4). In normal conditions, the activation of these receptors through ligand binding leads to homo- or hetero-dimerization, auto-phosphorylation, and the further phosphorylation and activation of downstream pathways implicated in cell growth and proliferation, such as Ras/MAPK, PI3K/Akt, or STAT signaling pathways. EGFR family proteins are frequently altered in some cancer entities (such as the lung, breast, brain, head and neck, colon, and pancreas) through gene amplification, protein overexpression, or activating mutations [63].

##### EGFR

Active EGFR mutants frequently found in non-small cell lung cancer (NSCLC; p.L858R, p.Δ746-750) bind to Beclin 1 at the endosome and phosphorylate it at three tyrosine residues (Y229, Y233, and Y352) [64]. Phosphorylated Beclin 1 dimerizes and is further inactivated through modifications in its interactome by increasing the Bcl-2 and Rubicon binding and preventing the binding of Vps34. Therefore, active EGFR leads to autophagy inhibition (Figure 1C). Treatment with the EGFR inhibitor Erlotinib results in autophagy induction as well as Beclin 1-tyrosine de-phosphorylation in sensitive EGFR-mutant NSCLC cells (HCC827), but not in resistant cells (H1975). The Beclin 1 phospho-mimetic mutant (Beclin 1 Y229/233/352E; Beclin 1 EEE) leads to autophagy suppression and a faster tumor growth in xenograft models as compared to the Beclin 1 wild-type control. Furthermore, *in vivo* xenograft studies on a tetracyclin-induced Beclin 1 EEE mutant indicate that these cells are more resistant to Erlotinib treatment compared to the control or wild-type Beclin 1 xenografts. Taken together, these data show that Beclin 1 tyrosine phosphorylation by oncogenic EGFR regulates autophagy and that it contributes to tumor progression [64]. 

Inactive EGFR has also been postulated as a regulator of autophagy through binding to LAPTM4B (lysosomal protein transmembrane 4b) and Sec5 (EXOC2, exocyst complex component 2), facilitating the binding of EGFR to Rubicon, competing with Beclin 1 binding to it, and therefore inducing autophagy [65]. Although this function of inactive EGFR has been proposed as playing a role in starvation conditions, no *in vivo* evidence has been provided so far. 

##### HER2

HER2 is a receptor-tyrosine kinase of the EGFR family that has no known ligand and that is frequently amplified in breast, bladder, and ovarian cancer [66]. HER2 has been shown to bind to Beclin 1 and to inhibit autophagy in HER2+ breast cancer cells [67,68]. Interestingly, it does not appear to phosphorylate Beclin 1, suggesting that it might alter the autophagy function through a Beclin 1-tyrosine phosphorylation-independent mechanism [68]. *In vivo* studies using knock-in mice containing a Beclin 1 mutation (Becn1^F121A^) that prevents its binding to its inhibitor Bcl-2 [69] (therefore showing increased basal autophagy in several tissues, including the mammary gland) indicated that increased basal autophagy abrogates mammary tumorigenesis in transgenic mice overexpressing HER2. Furthermore, treatment of HER2+ breast cancer xenografts with the autophagy-inducing peptide Tat-Beclin 1 [70] inhibits tumor growth as efficiently as the tyrosine kinase inhibitor Lapatinib does, disrupts the Beclin 1-HER2 complex, and induces a unique transcriptional profile [68].

#### 4.2.2. Akt

Akt (Protein kinase B) is a serine/threonine kinase that is found to be amplified and overexpressed in a number of cancers [71], and upstream mutations leading to its activation are frequently found in cancer. Activated Akt was shown to phosphorylate Beclin 1 at S234 and S295 [72], leading to Beclin 1 binding to 14-3-3 and vimentin and to further cytoskeleton sequestration and inactivation. Xenografts experiments of Rat2 fibroblasts transduced with a myristoilated-Akt version and either a vector control or a vector expressing Flag-Beclin 1 (wild-type or S234/295A) indicate that activated Akt was able to transform fibroblast to fibrosarcomas, that Beclin 1 wild-type overexpression prevents tumor growth, and that this effect is further enhanced by overexpressing the Beclin 1 S234/295A non-phophorylatable mutant. This tumorigenesis inhibition upon Beclin 1 overexpression (wild-type or S234/295A non-phosporylatable mutant) correlates with the induction of autophagy as determined by p62 levels detected by IHC in xenograft samples.

#### 4.2.3. CK1 and p300

Casein kinase 1 (CK1) family members are serine/threonine kinases that are ubiquitously expressed and that play different roles in regulating a variety of cellular processes, including apoptosis, proliferation, DNA repair, differentiation, and cellular trafficking [73]; furthermore, activating mutations and the overexpression of several CK1 isoforms have been implicated in cancer [74].

It was shown that CK1 phosphorylates Beclin 1 at residues T406 and S409. Phosphorylated Beclin 1 is then acetylated by p300 (E1A-binding protein, 300 kDa) at residues K430 and K437, and this reaction is reversed by SIRT1 (Sirtuin 1). *In vivo* experiments in MCF-7 cells demonstrated that acetylation at these two sites is required for tumorigenesis, since MCF-7 cells overexpressing Beclin 1 K430/437R mutation inhibit tumor growth to a larger extent than cells overexpressing Beclin 1 wild-type do. This effect correlates with decreased p62 and Ki67 levels, suggesting a higher autophagy activity and lower proliferation rates compared to the empty vector or the Beclin 1 wild-type controls [75].

#### 4.2.4. BCR-ABL

BCR-ABL fusion kinase (BCR: Breakpoint cluster region; ABL: Abelson murine leukemia viral oncogene homolog 1) is frequently found in chronic myeloid leukemia and some cases of acute lymphoblastic leukemia. The BCR-ABL fusion protein activity is deregulated and promotes the activation of oncogenic downstream pathways, leading to cell growth and proliferation and to the inhibition of apoptosis [76]. A recent report showed that BCR-ABL binds to Beclin 1 and phosphorylates it at Y233 and Y352, leading to its inactivation and autophagy inhibition [77]. Mice transplanted with bone marrow derived cells (BMDCs) depleted of Beclin 1 through a miR sequence showed an increased survival compared to the mice transplanted with the BMDCs expressing a miR control. This effect highlights the importance of Beclin 1 on a CML mouse model, although this experiment cannot ascertain whether the effect that is seen is due to changes associated with autophagy, Beclin 1 autophagy-independent functions or to the BCR-ABL-phosphorylated Beclin 1. Therefore, the significance of the BCR-ABL phosphorylation events on Beclin 1 *in vivo* remains unclear.

### 4.3. Other Beclin 1 Modifiers

Besides EGFR, HER2, and BCR-ABL, other tyrosine kinases, including RET (involved in acute myeloid leukemia) [78], have been implicated in regulating autophagy, but whether there is any effect in Beclin 1 is not known. 

Ubiquitination is also an important post-translational modification regulating Beclin 1, and there are excellent and extensive reviews available that address it [79,80].

## 5. Transcriptional Regulation of Beclin 1 and Its Relevance in Cancer

An additional level of regulation of Beclin 1 and autophagy activity is through the modulation of its transcript levels. Several miRNAs have been linked to the regulation of Beclin 1 levels, including miR-30a [81], miR-30d [82], miR-376b [83], and miR-519a [84], and, to a higher extent, autophagy modulation. However, their direct implications in cancer need to be explored.

Multiple transcription factors have also been linked to regulating Beclin 1 levels, including Jun [85], Forkhead box O (FOXO) family members FOXO1 and FOXO3 [86,87], Nuclear factor-kB (NFkB) [88], p63 [84], Signal Transducer and Activator of Transcription 1 (STAT1) [89], Peroxisome proliferator-activated receptor-α PPARα [90], X-box binding protein 1 (XBP-1) [91], and farnesoid X receptor FXR [90]. However, the relevance of this transcriptional regulation in cancer requires an extensive investigation.

## 6. Mouse Models of Beclin 1 Modification

*BECN1* is an essential gene for development, and *Becn1^−^*^/*−*^ mice die early in development due to a failure to cavitate the embryonic bodies [92]. Several mouse models of Beclin 1 alterations have been developed, including *Becn1^+/−^* mice. These mice show an increased susceptibility to tumor formation and an elevated incidence of lung carcinomas, lymphomas, hepatocellular carcinomas, and breast carcinomas, demonstrating the role of Beclin 1 in tumorigenesis [3,4]. 

*Becn1^+/−^* mice have been used to study several tumor entities: Subcutaneous melanoma xenografts showed increased hypoxia-induced angiogenesis in *Becn1^+/−^* mice as compared to the *Becn1^+/+^* mice [93]; T-cell lymphoma development in *Atm^−/−^* mice is delayed when crossed with *Becn1^+/−^* mice, although the effects in Eu-myc transgenic mice, a mouse model of Burkitt lymphoma [94], seems to accelerate the disease onset, highlighting the importance of examining each individual tumor type and stage; *Becn1^+/−^* mice also reduced *Tsc2^+/−^* tumorigenesis in the kidney [95]. Even though *Becn1^+/−^* mice showed an increased mammary tumor formation, the monoallelic loss of *Becn1* reduced *Palb2*-associated mammary tumorigenesis in a p53-dependent manner [96] in a mouse model of mammary tumorigenesis. Interestingly, *Becn1^+/−^* had no effect on *Erbb2*- or *PyMT*-driven mammary tumorigenesis [97].

A mouse model of increased autophagy was recently reported, where a whole-body knock-in mutation in *Becn 1*^F121A/F121A^ led to increased basal autophagy due to the decreased binding of Beclin 1 to its inhibitor Bcl-2 [69]. These mice are overall healthier than the wild-type mice and live longer. Crossing these mice with transgenic mice overexpressing HER2 in the mammary gland led to the suppression of mammary tumorigenesis, supporting the evidence that abrogating the inhibitory effect of HER2 in autophagy leads to the inhibition of tumor formation [68].

## 7. Conclusions

The role of autophagy in cancer has been extensively explored, although a full mechanistic picture remains to be established. Autophagy seems to play a protecting role against different stressors at the cancer initiation steps, although it may have a pro-tumoral activity for the same reason during the growth of the primary tumor, also providing fuel to the cells to keep the growth and proliferation rate. Therefore, it is of utmost importance to study whether promoting or inducing autophagy in specific backgrounds in the different tumor entities might be beneficial as a therapeutic option. Within this context, autophagy regulation by Beclin 1 has been shown to play a major role in tumorigenesis in several cancer types, and the molecular mechanisms underlying its effects are being elucidated. These studies might lead to important discoveries for Beclin 1 targeted therapies in cancer.

## Figures and Tables

**Table 1:** Beclin 1 modifications implicated in cancer.

Modified Residue	Modification	Enzyme	Effect on Autophagy	Tumor Type	Cell Type	Reference
Unknown		HER2	Inhibitory	Breast	BT474, SKBR3, MDAMB361, BT474VH2	[68]
S30	Phosphorylation	PGK1	Activating	Glioblastoma Multiforme	U87	[62]
S90	Phosphorylation	MK2/3	Activating	Breast	MCF-7	[25]
S90	Phosphorylation	DAPK3	Activating	Breast	MCF-7	[55]
S90	Phosphorylation	AMPK	Activating		MEFs	[58]
S93	Phosphorylation	AMPK	Activating		MEFs	[58]
T119	Phosphorylation	DAPK2	Activating		HEK293	[24]
Y229/233/352	Phosphorylation	EGFR	Inhibitory	Non-Small cell lung cancer	HCC827, H1975	[64]
Y233/352	Phosphorylation	BCR-ABL	Inhibitory	Leukemia	K562	[77]
S234/295	Phosphorylation	Akt	Inhibitory	Fibrosarcoma	Rat2	[72]
T388	Phosphorylation	AMPK	Activating		HEK293	[59]
T406/S409	Phosphorylation	CK1	Inhibitory	Breast	MCF-7	[75]
K430/437	Acetylation	p300	Inhibitory	Breast	MCF-7	[75]
